# LESA-MS detects differences in lipid profiles between healthy and footrot-affected tissues

**DOI:** 10.1039/d6an00560h

**Published:** 2026-07-14

**Authors:** Rebecca E. Greatorex, Sidrah Rahman, Kei F. Carver Wong, Thomas J. White, Catrin Rutland, Rachel Clifton, Rian L. Griffiths

**Affiliations:** a School of Pharmacy, University of Nottingham, University Park, University of Nottingham Nottingham NG7 2RD UK rian.griffiths@nottingham.ac.uk; b School of Life Sciences, University of Nottingham, University Park Nottingham NG7 2RD UK; c School of Veterinary Medicine and Science, University of Nottingham Sutton Bonington Campus Loughborough LE12 5RD UK

## Abstract

Ovine footrot is a highly contagious polymicrobial infection causing lameness in sheep with an associated UK economic burden upwards of £24m per annum. Changes in gene expression affecting lipid metabolism have been reported in footrot-affected compared to healthy feet, however, the role of lipids in footrot pathogenesis has not been studied. Previously, liquid extraction surface analysis mass spectrometry (LESA-MS) has been used to sample lipids from bacterial colonies and thin tissue sections. Here we present LESA-MS analysis of lipids extracted from both healthy and footrot-affected tissue samples. The samples comprised abundant phospholipids in the region *m*/*z* 700–850, that were common to both sample types, two of which were identified as phosphatidylcholines PC 16:0/18:1 and PC 16:0/16:1. Abundant lipids in each sample between *m*/*z* 950–1150 were different. A variety of lipids identified by accurate mass and MS/MS as triacylglycerols were higher in abundance in healthy tissue sections than footrot-affected ones, with *t*-tests confirming statistical significance. Lipids identified by accurate mass as either phosphatidylserines or phosphatidylthreonines were detected in higher abundance in footrot-affected tissue sections; MS/MS did not aid assignment of these. We present a rapid lipidomic analysis approach that could inform on the role of lipids in pathogenesis of footrot and other inflammatory diseases.

## Abstract 2

This study looked to use a rapid approach for tissue biopsy lipidomics to investigate differences between heathy and ovine footrot tissues.

## Graphical abstract

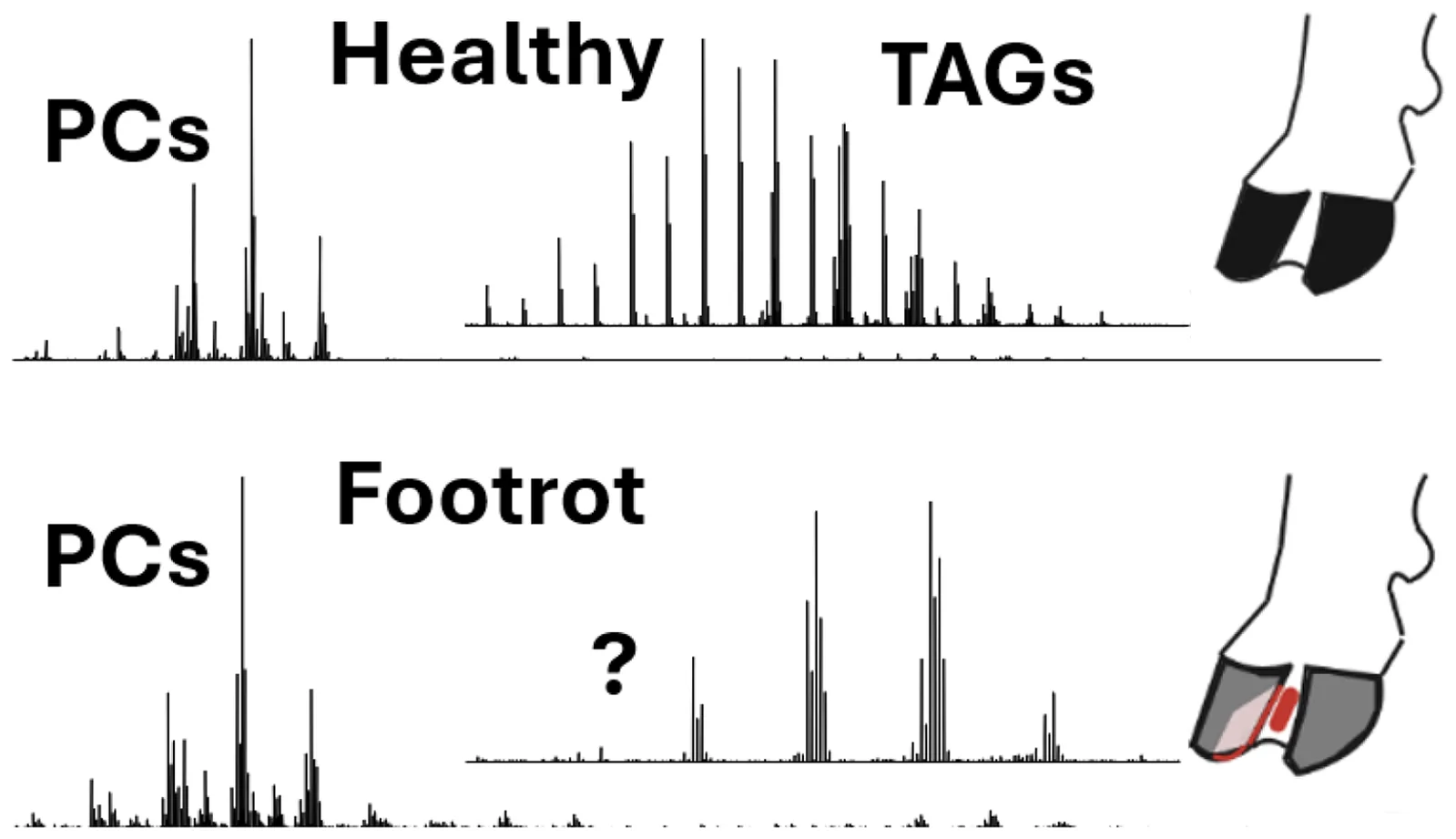

## Introduction

Ovine footrot is a highly contagious polymicrobial disease affecting the interdigital skin of sheep and is the main cause of lameness in sheep globally.^1^ Footrot is a significant concern for animal welfare due to the pain associated with foot lesions. The disease also has economic impacts, with costs of ∼£24–£80m per year in the UK alone.^2,3^ The primary causative bacterium is *Dichelobacter nodosu*s (*D. nodosus*),^4^ which is present on 99% of UK farms.^5^ Whilst *D. nodosus* is essential for footrot to occur, footrot is known to be a polymicrobial infection. *Fusobacterium necrophorum*, *Porphyromonas asaccharolytica*, and *Mycoplasma fermentans* are associated with footrot.^6–8^

The pathogenesis of footrot is complex, and risk factors include poor hygiene, warm and damp environmental conditions, injury to interdigital skin, host genetic factors and dysbiosis of the interdigital skin microbiota, with a combination of factors required to initiate disease.^1,6,9^ Disease can progress from mild dermatitis to complete separation of hoof horn and tissue necrosis.^4^ The local host immune response is an important component of disease pathogenesis, with increased expression of pro-inflammatory cytokines TNFα and IL1β and pattern recognition receptors TLR2 and TLR4 in the interdigital skin during disease.^10^ Increased expression of these inflammatory mediators is followed by infiltration of immune cells into the epidermis.^10^

Lipids and their metabolites are potent regulators of inflammation and other cell signalling cascades,^11^ and there is preliminary evidence of changes in lipid metabolism during footrot, including upregulation of genes involved in fatty acid synthesis, and downregulation of fatty acid metabolic processes.^7^ Lipids also play an important role in maintaining skin barrier function^12^ and given that loss of integrity of the skin barrier is required for bacterial invasion in footrot, lipids may be relevant for understanding the initial stages of disease. However, to date there have been no studies of the role(s) of lipids in pathogenesis of footrot. Lipid function is dependent on cellular localisation,^13,14^ therefore spatial lipidomics can inform on localisation of lipids as well as providing quantitative and qualitative information.^15^

Mass spectrometry (MS) offers an untargeted analytical approach suitable for the extraction of various analytes without the need for chemical tagging or modification.^16^ Surface analysis MS offers a rapid, high throughput and untargeted approach for biomolecule analysis from surfaces including tissue sections and biofilms. Several such approaches have been used to directly extract biomolecules such as proteins,^16,17^ lipids^18–20^ and metabolites,^19,21,22^ directly from biological samples. Bacterial speciation can be achieved from cultures grown from swabs of *e.g.* clinical isolates cultured from lung sputum^23,24^ using surface analysis MS approaches.

Liquid extraction surface analysis mass spectrometry (LESA-MS) involves automated sampling *via* a liquid junction formed between the sample surface and a robotically operated pipette tip; extracts are then directly (nano) electrosprayed *via* this conductive pipette tip. Solvent systems can be tailored according to the analyte of interest, to maximise extraction and analysis time. LESA-MS has enabled the study of bacterial proteins,^17^ lipids^20^ and metabolites,^25^ including those from polymicrobial biofilms. Infected samples, such as bacterially infected wound models, have previously been interrogated *via* LESA-MS, with different protein profiles observed between non-infected and infected samples.^26^ However, naturally bacterially infected tissues have not routinely been analysed. The sensitivity afforded by LESA-MS, particularly for MS/MS assignments, will be exploited from biopsies.

This study looks to determine the feasibility of profiling lipidomic signatures from ovine interdigital skin biopsies, performing direct lipidomic analysis of ovine tissue biopsies for the first time. We also report preliminary findings from comparing healthy lipidomic profiles with those from footrot-affected tissue samples. We demonstrate similarities in phosphatidylcholine profiles between healthy and footrot-affected samples, confirmed *via* MS/MS experiments. Moreover, key differences between sample types are demonstrated in the region *m*/*z* 950–1150 with triacylglycerol lipids detected in higher abundance in healthy tissue samples, assigned by MS/MS experiments. Yet different lipids were detected in greater abundance in the same *m*/*z* region in footrot samples. The methodology presented herein paves the way for larger scale studies using LESA-MS to understand pathogenesis of complex polymicrobial diseases such as footrot.

## Experimental

**Fig. 1:**
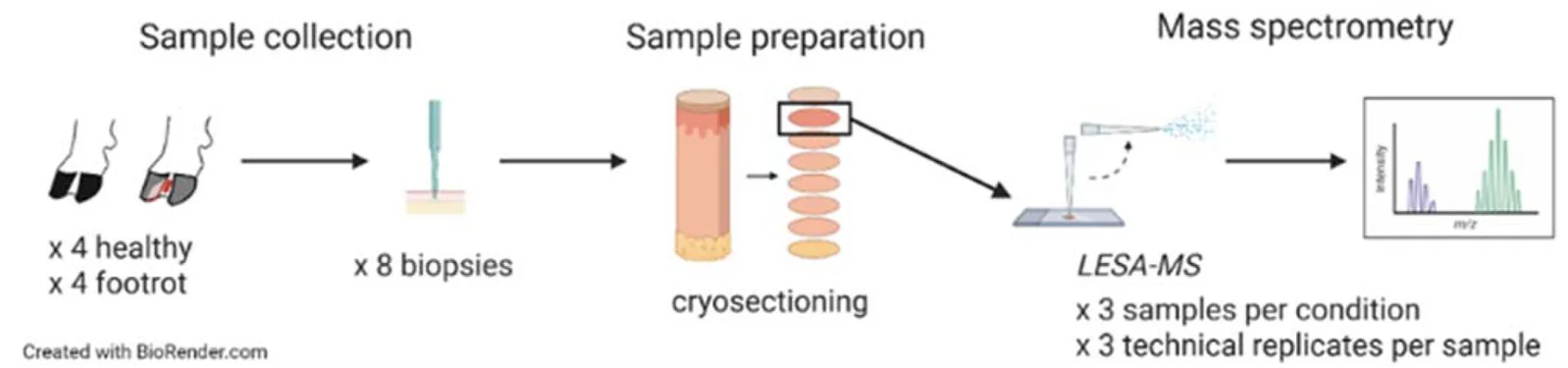
A representation of the workflow for this study. Tissue biopsies were taken from four healthy and four footrot affected sheep before cryosectioning onto slides and performing LESA-MS.

Biopsies were obtained post-mortem from lambs at a UK abattoir using a convenience sampling approach; all samples were collected on one day in April 2022. Information was not available on the individual sheep or flocks from which the samples originated. Sheep feet were assessed for presence of footrot lesions according to a recognised scoring system.^27^ Biopsies were collected from the interdigital skin of four healthy and four footrot-affected feet using a 6 mm punch (Qiagen GmbH, Hilden, Germany) and immediately placed into phosphate buffered formalin. Transverse cryosectioning was performed by encasing biopsies in water on individual metal plates, ensuring no contact with any other materials, and freezing at −20 °C. Each block was sectioned at 30 µm using a cryostat (Leica Biosystems, Germany) set at −20 °C. Serial transverse sections of each biopsy were cut using a N35 long duration stainless steel microtome blade (FEATHER®, Osaka, Japan). The cryostat blade and surrounding area were cleaned with 70% ethanol then distilled H_2_O prior to cutting each new section to prevent potential bacterial contamination. Each section was placed on a non-coated microscope slide; these were maintained at −20 °C until further analysis. The overall workflow is detailed in Fig. 1.

Three of the four biological replicates from each sample type were analysed using LESA-MS. Background extraction solvent spectra were also obtained. LESA was carried out on each tissue section using the Triversa Nanomate (AdvionBiosciences, Ithaca, NY, USA), in triplicate (technical replicates). After initial analysis of the first and then second section (from the skin surface) subsequent samples focused on the second section from each biological repeat. The extraction/ionisation solvent was 2 : 1 chloroform : methanol. During extraction, 10 μL of solvent was aspirated from the solvent well, before sampling the tissue with 2 μL of this solvent for 1 second. Finally, 2.5 μL of the sampling solvent was re-aspirated and infused into the mass spectrometer at a gas pressure of 0.3 psi and a potential of 1.7 kV.

Experiments were performed on a Thermo Fisher Orbitrap Q-Exactive instrument in positive ionisation mode, to afford structurally informative [M + Na]^+^ adducts for MS/MS. Spectra were recorded in full scan mode at a resolution of 140 000 at *m*/*z* 400 from *m*/*z* 80–1200. The AGC target was 1 × 10^6^ charges, maximum injection time 500 ms. Tandem MS/MS experiments *via* high-energy collision-induced dissociation (HCD) were performed with an AGC target of 1 × 10^6^ charges, maximum injection time of 50 ms, at a resolution of 35 000, normalised collision energy 10–35%, isolation width *m*/*z* 1.0, and 1 microscan. MS and MS/MS data were recorded for 1 minute and analysed using Thermo Xcalibur software version 4.2.28.14. MS/MS spectra were interpreted manually. Lipids were assigned by searching *m*/*z* peaks against the LIPIDMAPS database^28^ using the following criteria: 5 ppm, protonated, sodium and potassium adducts.

Statistical analysis was performed using MetaboAnalyst to conduct univariate statistics, *via* nonparametric Mann–Whitney test, and multivariate principal component analysis (PCA). Peak intensity data was normalised to the lipid fraction, log_10_ transformed and pareto scaled to preprocess the data for PCA. Statistical significance was determined based on *p*-values where values above 0.05 were nonsignificant (ns) and values below 0.05 were significant; *p-value < 0.05, **p-value < 0.01. MetaboAnalyst was also used to generate volcano plots and PCA plots to visualise lipidomic differences. Data was visualised using GraphPad Prism to plot bar charts.

## Results and discussion

### Detection of lipids from ovine tissues using LESA-MS

Previously, LESA has been used for the analysis of metabolites and lipids from tissue samples^29^ and bacterial colonies,^20,25^ furthermore compatibility with formalin fixation including phosphate buffered fixatives has been demonstrated.^29,30^ Healthy and footrot-affected formalin fixed tissue sections were sampled with 2 : 1 chloroform : methanol *via* LESA-MS to target analysis of lipids. From the first section taken from the outer region of the foot, a high polymer signal was detected, however this was not the case from the second serial section. Subsequent repeats focused on the second serial section in each case. A high lipid signal intensity was detected and one minute of data collection was sufficient. Most ions were assigned (*via* accurate mass) as sodium adducts, within 5 ppm of the calculated mass, which is expected when analysing formalin fixed tissue samples.^30^ Here we present a rapid method for the analysis of tissue biopsy lipids; our approach is a rapid alternative in comparison to alternative monitoring methods suggested in the literature such as PCR and microbial culture from swab analysis.^31,32^

### Common lipids in healthy and footrot samples

**Fig. 2:**
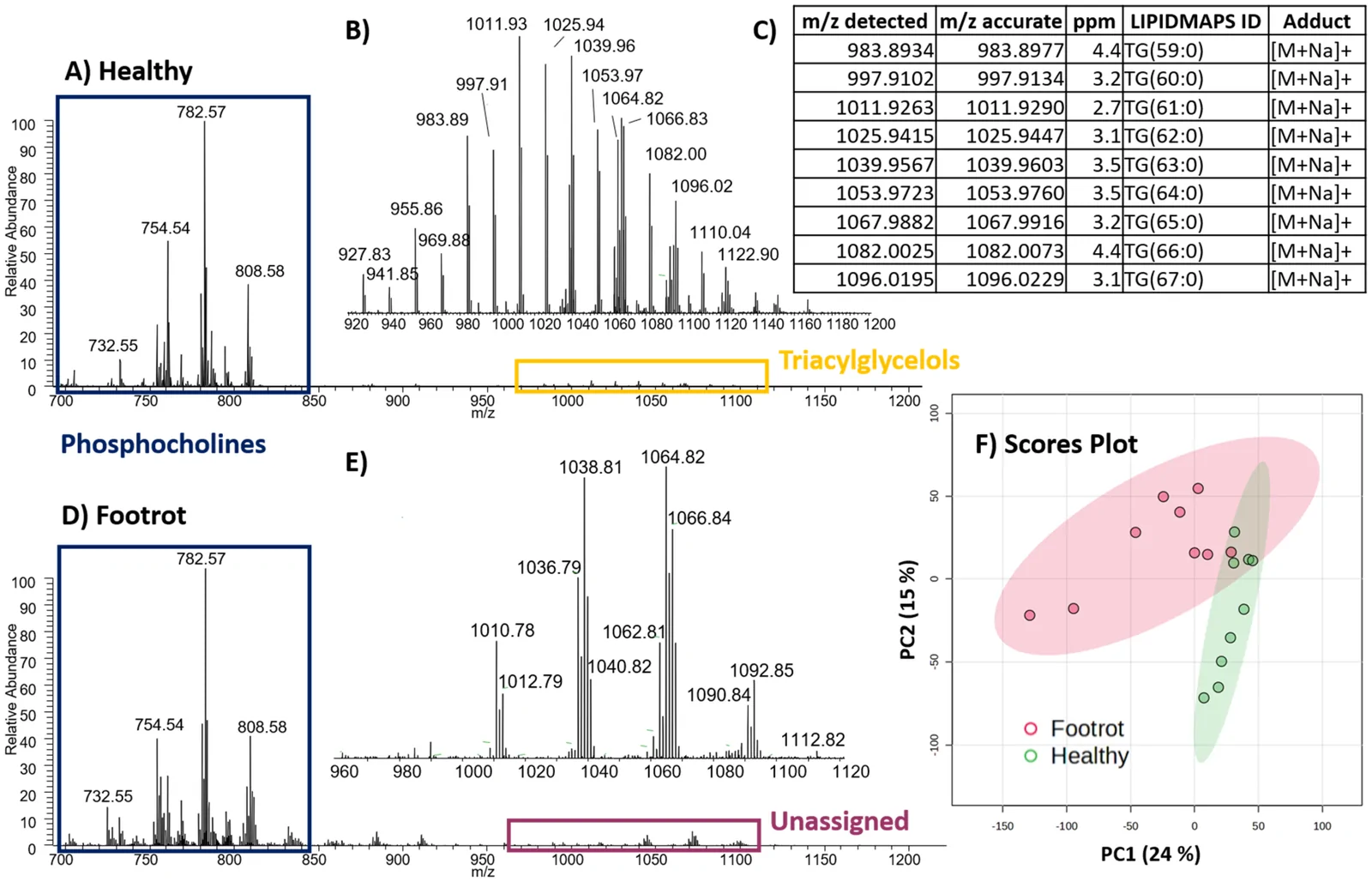
LESA-MS spectra following sampling of (A) healthy tissue with detected phospholipids highlighted in the region *m*/*z* 700–850. An inset of higher *m*/*z* lipids assigned as triacylglycerols is shown in panel B, and a table detailing these lipids in panel C. The *m*/*z*'s highlighted were detected in biological and technical replicates. Spectra obtained from sampling (D) footrot tissue with detected phospholipids highlighted in the region *m*/*z* 700–850. An inset of unassigned higher *m*/*z* lipids is shown in panel E, and a PCA plot of the two sample types in panel F.

**Fig. 3:**
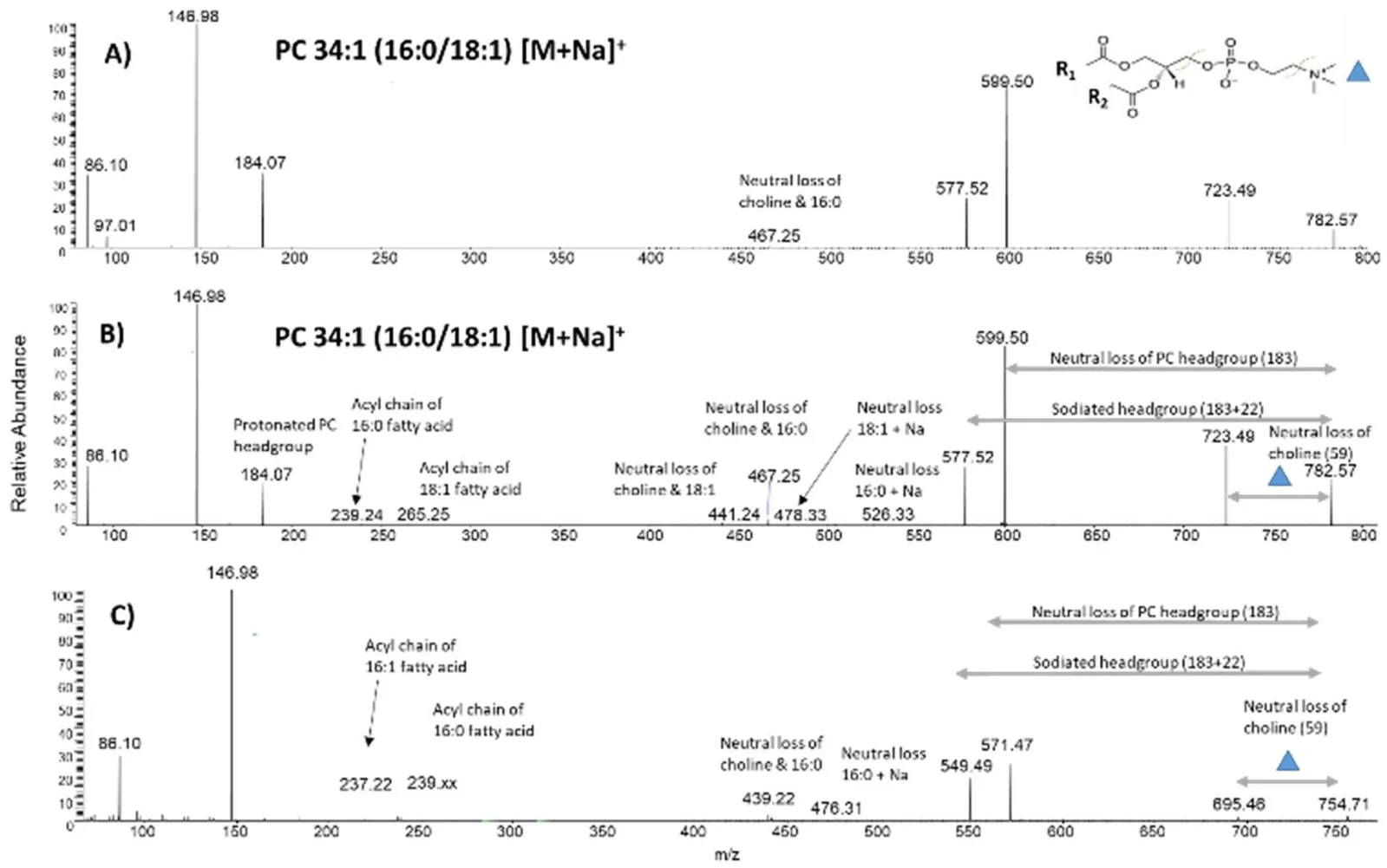
LESA-MSMS spectra following high-energy collision-induced dissociation (HCD) of (A) *m*/*z* 782.57 from healthy tissue, (B) *m*/*z* 782.57 from footrot tissue and (C) *m*/*z* 754.54 from healthy tissue. These lipids were abundant in both sample types and are assigned as phosphatidylcholine lipids.

Using the 2 : 1 chloroform : methanol extraction solvent, abundant ions were detected in the region *m*/*z* 700–850 in each sample type, in the phospholipid region, see Fig. 2A and D. Abundant ions common to each sample include *m*/*z* 732.55, 754.54, 782.57 and 808.58. These species can be assigned as a mixture of protonated and sodium adducts of phospholipids based on accurate mass, see SI Table 1. The most abundant ion was *m*/*z* 782.5659; upon high-energy collision-induced dissociation (HCD) several product ions characteristic of phosphatidylcholine were detected from each sample, see Fig. 3. The neutral loss of 59 (choline), the neutral loss of 183 (phosphatidylcholine), and a phosphate headgroup fragment at *m*/*z* 146.98 that is indicative of a sodium adduct. Neutral losses indicative of the fatty acid side chains was also detected; neutral loss of palmitic acid (256, 16:0), with and without sodium, neutral loss of oleic acid (282, 18:1) with sodium, the further loss of choline and acyl ions of each of these chains at *m*/*z* 239 and 265 respectively, see Fig. 3. Hence this lipid can be assigned as PC 16:0/18:1. Similar PC headgroup losses and ions were observed upon HCD of *m*/*z* 754.5437, see Fig. 3. Fatty acid side chain acyl ions at *m*/*z* 237 and 239, alongside neutral losses of palmitic acid (256, 16:0, and further loss of sodium, and choline) and the neutral loss of 254, 16:1, and further loss of sodium, and choline, suggest that the major component of this lipid is PC 16:0/16:1. Phosphatidylcholine lipids are common components of mammalian cell membranes, it is perhaps therefore unsurprising that this lipid class was so abundant in both healthy and footrot samples. Also, oleic and palmitic acid fatty acids have previously been described as major components of ovine cell membranes.^33^*Myco-plasma* spp. have been implicated previously in footrot;^7^ phospholipids have also been indicated (unusually) in these bacterial cell walls.^34^ Here we show that LESA-MS can be used for rapid and direct analysis and structural characterisation of phospholipids from footrot tissue samples.

**Fig. 4:**
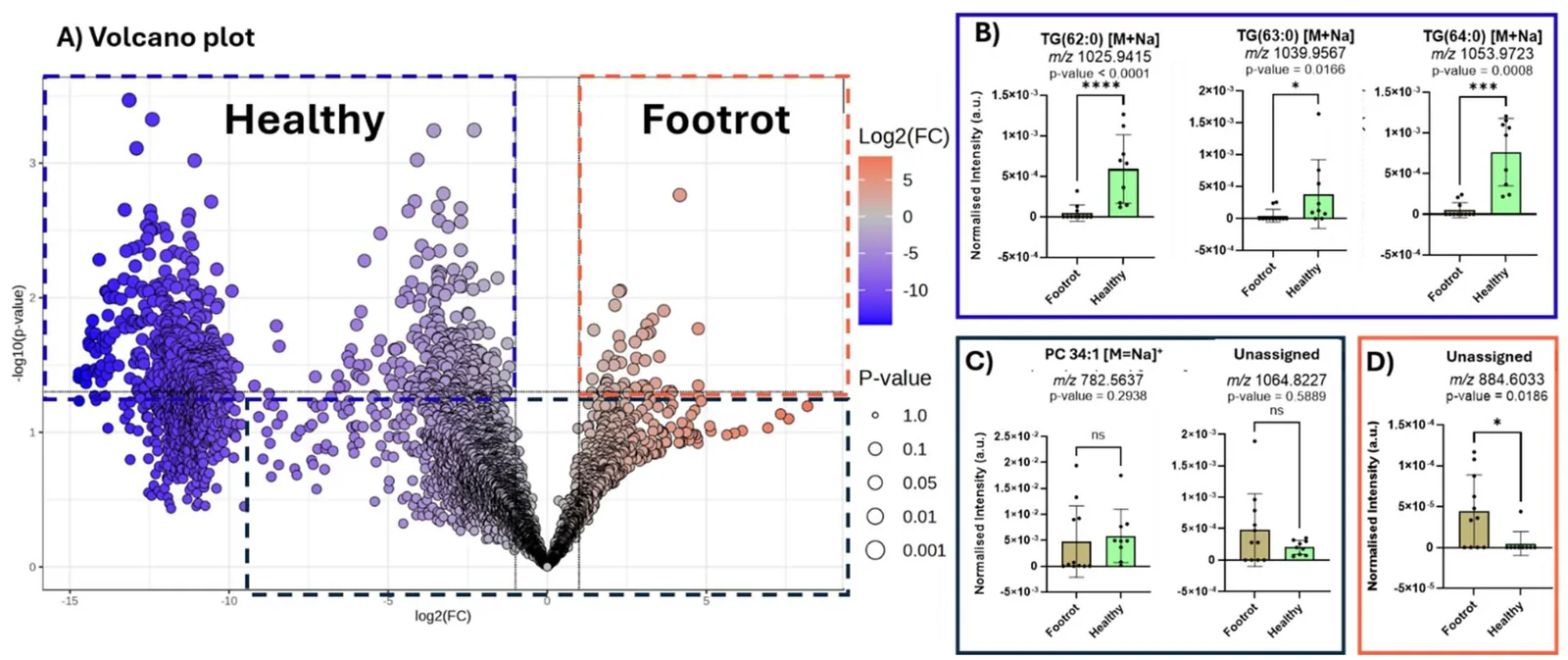
(A) Volcano plot based on lipidomic data generated from LESA-MS of healthy and footrot-affected tissue biopsies showing that detected lipid were reduced in footrot-affected samples. The volcano plots were generated using nonparametric Mann–Whitney test with a fold-change threshold of 2 and *p*-values below 0.05; ** *p*-value < 0.01. *P*-Values above 0.05 were found to be not significant (ns). Bar charts show example lipids statistically (B) significantly elevated in healthy biopsies and (C) no difference and (D) significantly elevated in footrot biopsies.

Lipids that show no statistically significant difference can be visualised in the black coloured box in the volcano plot in Fig. 4A. The phosphatidylcholine lipids discussed above contributed to this profile. Bar charts alongside *t*-tests confirm no statistically significant difference, with *p* values for PC (16:0/18:1) of 0.2938, Fig. 4C. The two samples show a high degree of similarity upon principal component analysis, Fig. 2F.

### Abundant lipids in healthy samples

Abundant ions were also detected in the region *m*/*z* 950–1150 in each tissue type, see Fig. 2B & E. However, based on accurate assignments using the LIPIDMAPS database, different lipids were most abundant in each sample. In the healthy tissue samples, a total of nine lipids were particularly abundant; assigned based on accurate mass (within 5 ppm) as sodium adducts of saturated triacylglycerols (TG) ranging from TG (59:0) up to (67:0) in this mass range, see Fig. 2C. The following ions were detected reliably across three biological repeats: 983.89, 997.91, 1025.94, 1053.97, 1082.00. The ion at *m*/*z* 1011.93 was detected reliably across two biological repeats. These contribute to the significantly different signals displayed in the blue coloured box in the volcano plot in Fig. 4A. Bar charts confirm this, with *p* values for TG (62:0), (63:0), and (64:0) of <0.0001, 0.0016 and 0.0008 respectively (Fig. 4B). Additional lipid ions detected in this mass range were assigned *via* accurate mass (within 5 ppm), see SI Table 2.

**Fig. 5:**
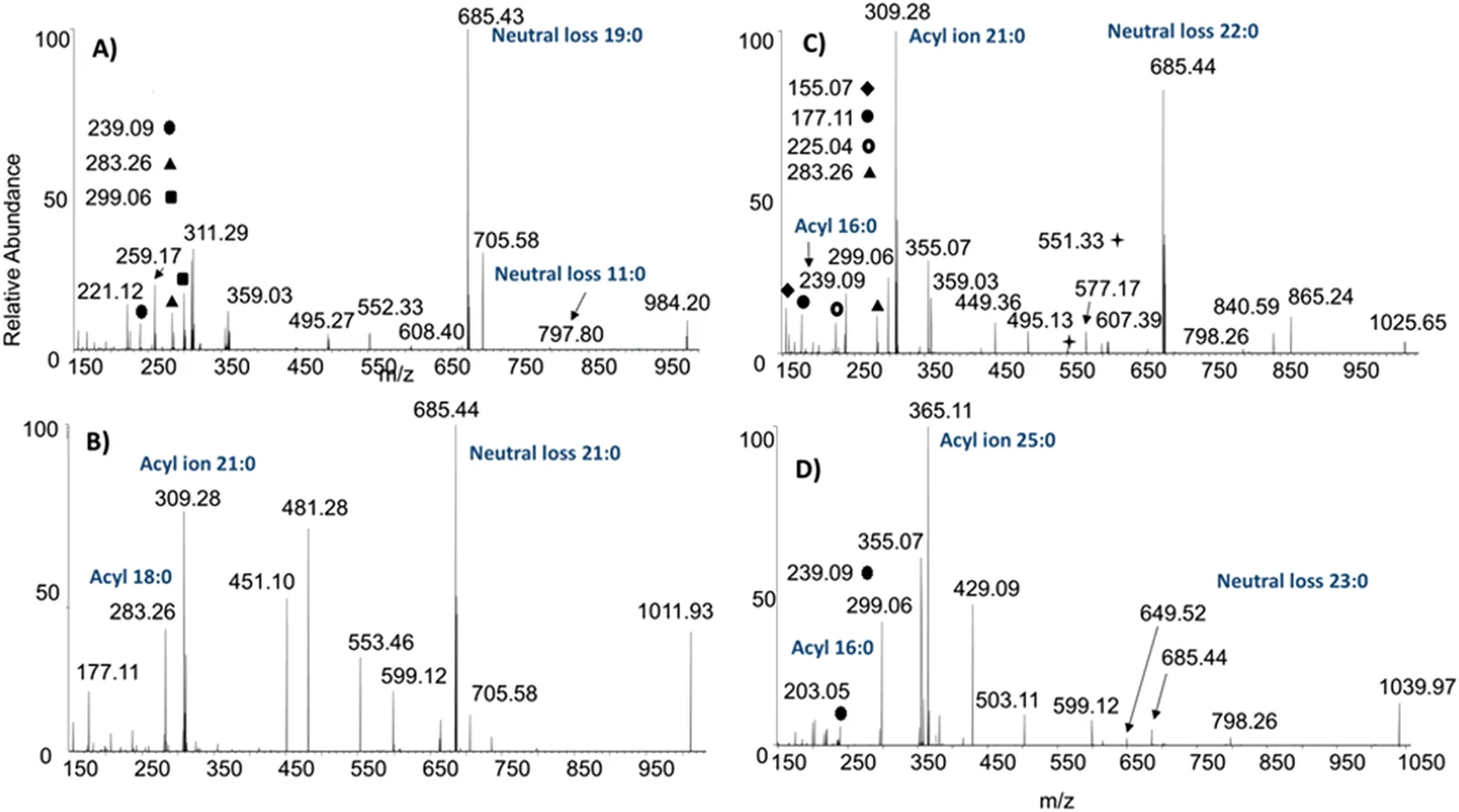
LESA-MSMS spectra following high-energy collision-induced dissociation (HCD) of (A) *m*/*z* 983.80 at CE 30, (B) *m*/*z* 1011.90 at CE 20, (C) 1025.90 at CE 25 and (D) *m*/*z* 1039.90 at CE 20 from healthy tissue. These lipids were more abundant in healthy tissue sections and are assigned as triacylglycerol lipids.

HCD of a range of the above-described ions was performed as shown in Fig. 5. Firstly, neutral loss of 298 (*m*/*z* 685) and 186 (*m*/*z* 797) from *m*/*z* 983.80 suggest fatty acid side chain components of 19:0 and 11:0 upon dissociation suggesting this TG comprises TG (19:0, 19:0, 11:0). HCD of *m*/*z* 1011.90 indicated neutral losses of 326 (*m*/*z* 685), 306 (*m*/*z* 705), along-side acyl ions at *m*/*z* 309 and 283 suggesting 21:0, 18:0 and 22:0 fatty acid side chains. HCD of *m*/*z* 1025.90 indicated the neutral loss of 340 (*m*/*z* 685), alongside acyl ions at *m*/*z* 309, 283 and 239 suggesting fatty acid side chain components ranging 22:0, 21:0, 18:0 and 16:0 respectively, suggesting a mixture of lipids contributing to TG 62:0.

These TG lipids were detected in both healthy and footrot samples, suggesting that they are present in either the sheep tissue, or both eukaryotic and bacterial components. TGs were detected in up to a ∼ten-fold higher abundance in healthy tissue in comparison to footrot tissue sections, see bar charts in Fig. 4B–D. Similarly, TGs have been shown to be reduced in other inflammatory conditions including periodontitis^35^ and atopic dermatitis.^36^ TGs contribute to skin barrier function^12^ therefore reduction in their abundance in footrot may contribute to disease pathogenesis. The saturated nature of these abundant lipids is noted, particularly in comparison to the mixed saturated/unsaturated nature of abundant lipids in footrot tissue (see below). Although further validation is required, this is promising evidence that our LESA method offers a route to investigating the role of lipids in disease pathogenesis.

### Abundant lipids in footrot-affected samples

In the footrot-affected tissue samples, the region *m*/*z* 950–1150 was dominated by ions assigned *via* accurate mass in LIPID-MAPS (within 5 ppm) as a mixture of saturated and unsaturated lipids with up to three double bonds and as either phosphatidylserines (PS), carbon numbers 50–56, or phosphatidylthreonines (PT), carbon numbers 49–53. These remain tentatively assigned as attempts to identify them *via* MS/MS were unsuccessful. Although these were detected between a two- and five-fold increase in footrot-affected samples in comparison to healthy samples, *t*-tests showed no statistically significant difference, see Fig. 4C. PS lipids are found in both eukaryotic and prokaryotic cells and play a key role in apoptosis (cell death).^37^ Although some signals were statistically significantly different, see Fig. 4D, more work is needed to identify these lipid signatures. As evidenced in the volcano plot (Fig. 4A) an overall reduction in lipid signatures was evident in the footrot-affected tissue, using our direct and rapid biopsy lipidomics approach. Although this is a small sample size and further validation is required, this is promising evidence that disease specific lipids could offer useful insights into disease pathogenesis.

## Conclusions

We demonstrate LESA-MS to study lipid profiles from footrot-affected tissues for the first time. Furthermore, we show that lipidomic analysis from healthy and footrot-affected tissue biopsies could yield new insights regarding disease pathogenesis. Our study demonstrates that there are distinct lipid differences between healthy and footrot samples, namely higher abundances of triacylglycerols in healthy tissue in the region *m*/*z* 950–1100 and unassigned lipids in footrot tissue. Overall, lipid signals were reduced in footrot-affected tissues, evidencing dysbiosis. Future work will include negative ion mode data acquisition and focus on identifying the bacterial strains present in footrot tissue samples, including further mass spectrometry analysis of these strains, *e.g.* MS/MS assignments of additional lipids, and additional analytes such as bacterial metabolites, as well as more biological repeats for future tissue studies.

## Author contributions

Conceptualization, R. L. G. and R. C.; methodology, R. L. G., C. R., S. R., R. E. G. and R. C.; formal analysis, K. F. C. W., T. W. and R. L. G.; resources, R. L. G, R. C. and C. R.; data curation, R. L. G.; writing—original draft preparation, R. L. G.; writing—review and editing, R. L. G., K. F. C. W., R. C. and C. R.; visualization, R. L. G. and R. C.; supervision, R. L. G.; project administration, R. L. G. and R. C. All authors have read and agreed to the published version of the manuscript.

## Conflicts of interest

There are no conflicts to declare.

## Supplementary Material

AN-151-D6AN00560H-s001

## Data Availability

Raw mass spectrometry data files can be accessed *via* the University of Nottingham repository (https://doi.org/10.17639/nott.7526). The supporting data has been provided as part of the supplementary information (SI). Supplementary information: Fig. S1 MS/MS spectra, and Tables S1 and S2 detected lipids. See DOI: https://doi.org/10.1039/d6an00560h.
