# Supplementary material for: LESA-MS detects differences in lipid profiles between healthy and footrot-affected tissues
Source: Analyst. 2026 Jul 14. Online ahead of print. doi: 10.1039/d6an00560h (PMC13390787; doi:10.1039/d6an00560h)
Supplement: AN-OLF-D6AN00560H-s001 [file AN-OLF-D6AN00560H-s001.pdf]

Supplementary Information (SI) for Analyst.
This journal is © The Royal Society of Chemistry 2026

LESA-MS detects differences in lipid profiles between healthy and footrot-affected tissues

Rebecca E. Greatorex[a], Sidrah Rahman[a], Kei. F. Carver Wong[a], Thomas J. White[b], Catrin Rutland[c], Rachel Clifton[c] and

Rian L. Griffiths[a†]

[a] School of Pharmacy, University of Nottingham, University Park, University of Nottingham, Nottingham, United Kingdom, NG7 2RD.

[b] School of Life Sciences, University of Nottingham, University Park, Nottingham, UK, NG7 2RD.

[c] School of Veterinary Medicine and Science, University of Nottingham, Sutton Bonington Campus, Loughborough, UK, LE12 5RD.

[†]Corresponding author: rian.griffiths@nottingham.ac.uk

Supplemental Figure

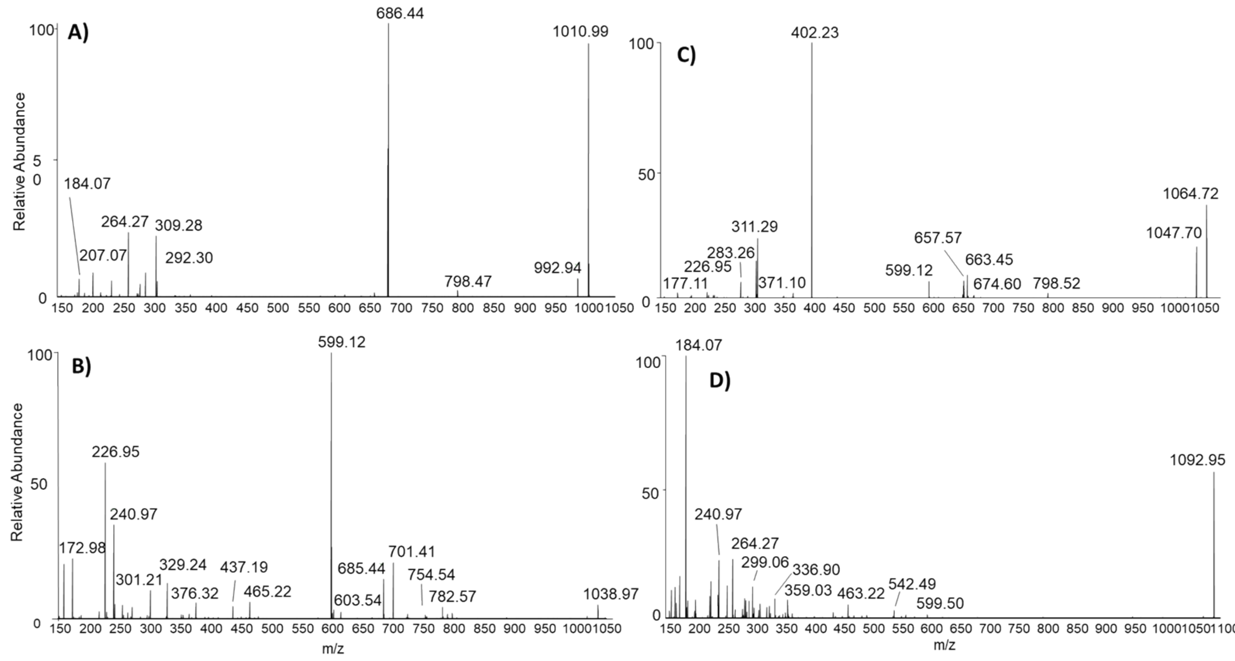

Supplemental Figure 1. LESA-MSMS spectra following High-energy Collision-induced dissociation (HCD) of ions from footrot tissue. HCD of A) m/z 1010.80 at CE 10, B) m/z 1038.80 at CE 20, C) m/z 1064.80 at CE 10, and D) m/z 1092.80 at CE 35. These lipids were more abundant in footrot tissue sections and were assigned as either PS or PT lipids according to accurate mass. Characteristic PS ions were not detected.

Supplemental Tables

Lower Mass Range

Supplemental Table 1 Lipid annotations from LIPIDMAPS in the region m/z 700-950.

Input Mass	Matched Mass	ppm	Assignment	Adduct	Healthy	Footrot
700.6560	700.6578	-2.6	Cer 44:1;O2	[M+Na]+	☑	☑
703.5732	703.5748	-2.3	SM 34:1;O2	[M+H]+	☑	☑
703.5732	703.5748	-2.3	CerPE 37:1;O2	[M+H]+	☑	☑
704.6144	704.6163	-2.7	Cer 42:1;O4	[M+Na]+	☑	☑
706.5370	706.5381	-1.6	PC 30:0	[M+H]+	☑	☑
706.5370	706.5381	-1.6	PE-NMe 32:0	[M+H]+	☑	☑
713.4331	713.4364	-4.6	PG 30:2	[M+Na]+	☑	☐
716.6517	716.6527	-1.4	Cer 44:1;O3	[M+Na]+	☑	☐
725.5550	725.5568	-2.5	SM 34:1;O2	[M+Na]+	☑	☑
725.5550	725.5568	-2.5	CerPE 37:1;O2	[M+Na]+	☑	☑
727.4552	727.4521	4.3	PG 31:2	[M+Na]+	☑	☐
727.4552	727.4545	1.0	PG 33:5	[M+H]+	☑	☐
727.5712	727.5724	-1.6	SM 34:0;O2	[M+Na]+	☑	☐
728.4523	728.4497	3.6	PS 32:4	[M+H]+	☑	☐
728.5182	728.5201	-2.6	PC 30:0	[M+Na]+	☑	☑
728.6876	728.6891	-2.1	Cer 46:1;O2	[M+Na]+	☑	☑
730.5354	730.5381	-3.7	PC 32:2	[M+H]+	☑	☑
732.5521	732.5538	-2.3	PC 32:1	[M+H]+	☑	☑
732.6463	732.6476	-1.8	Cer 44:1;O4	[M+Na]+	☑	☑
734.5660	734.5694	-4.6	PC 32:0	[M+H]+	☑	☑
743.6094	743.6061	4.4	CerPE 40:2;O2	[M+H]+	☑	☑
743.6094	743.6103	-1.2	CE 22:2	[M+K]+	☑	☑
746.5675	746.5694	-2.5	PC 33:1	[M+H]+	☑	☑
752.5178	752.5201	-3.1	PC 32:2	[M+Na]+	☑	☑
754.5339	754.5357	-2.4	PC 32:1	[M+Na]+	☑	☑
756.5495	756.5514	-2.5	PC 32:0	[M+Na]+	☑	☐
703.6348	756.7204	-2.2	Cer 48:1;O2	[M+Na]+	☑	☑
758.5677	758.5694	-2.2	PC 34:2	[M+H]+	☑	☑
760.5839	760.5851	-1.6	PC 34:1	[M+H]+	☑	☑

760.6780	760.6789	-1.2	Cer 46:1;O4	[M+Na]+	☑	☐
767.5381	767.5351	3.9	PA O-39:2	[M+K]+	☑	☐
768.5496	768.5514	-2.3	PC 33:2	[M+Na]+	☑	☑
769.5529	769.5508	2.7	PA O-39:1	[M+K]+	☑	☐
771.4743	771.4725	2.3	PA O-40:7	[M+K]+	☑	☐
772.5826	772.5851	-3.2	PC 35:2	[M+H]+	☑	☑
774.5985	774.6007	-2.8	PC 35:1	[M+H]+	☑	☑
778.5337	778.5357	-2.6	PC 34:3	[M+Na]+	☑	☑
780.5498	780.5514	-2.0	PC 34:2	[M+Na]+	☑	☑
782.5656	782.5670	-1.8	PC 34:1	[M+Na]+	☑	☑
786.5991	786.6007	-2.0	PC 36:2	[M+H]+	☑	☑
790.5340	790.5357	-2.2	PC 35:4	[M+Na]+	☑	☑
792.5494	792.5514	-2.5	PC 35:3	[M+Na]+	☑	☑
806.5646	806.5670	-3.0	PC 36:3	[M+Na]+	☑	☑
808.5809	808.5827	-2.2	PC 36:2	[M+Na]+	☑	☑
815.5029	815.5045	-2.0	PI O-32:2	[M+Na]+	☑	☐
815.5029	815.5069	-4.9	PI O-34:5	[M+H]+	☑	☐
836.6112	836.6140	-3.3	PC 38:2	[M+Na]+	☑	☑
836.6112	836.6140	-3.3	PE 41:2	[M+Na]+	☑	☑
838.6264	838.6296	-3.8	PC 38:1	[M+Na]+	☑	☑

Higher Mass Range

Supplemental Table 2 Lipid annotations from LIPIDMAPS in the region m/z 920-1200.

Detected m/z	Matched m/z	ppm	Assignment	Ion	Healthy	Footrot
**923.6931**	923.6947	1.7	PI O-41:0	[M+H]+		☑
**924.5891**	924.5879	1.3	PC 44:8	[M+K]+	☑	
**925.8184**	925.8195	1.2	TG 55:1	[M+Na]+		☑
**927.8349**	927.8351	0.2	TG 55:0	[M+Na]+	☑	☑
**935.8037**	935.8038	0.1	TG 56:3	[M+Na]+	☑	
**939.8334**	939.8351	1.8	TG 56:1	[M+Na]+		☑
**941.8468**	941.8508	4.2	TG 56:0	[M+Na]+	☑	☑
**947.5621**	947.562	0.1	PI 41:6	[M+Na]+	☑	
**953.8495**	953.8508	1.4	TG 57:1	[M+Na]+		☑
**955.8627**	955.8664	3.9	TG 57:0	[M+Na]+	☑	☑
**966.7416**	966.7369	4.9	IPC 46:0;O3	[M+H]+	☑	
**967.865**	967.8664	1.4	TG 58:1	[M+Na]+		☑
**969.8782**	969.8821	4.0	TG 58:0	[M+Na]+	☑	☑
**975.4741**	975.4714	2.8	DGDG 34:8;O2	[M+K]+	☑	
**979.5756**	979.5755	0.1	DGDG 36:4	[M+K]+	☑	
**981.8815**	981.8821	0.6	TG 59:1	[M+Na]+		☑
**983.8934**	983.8977	4.4	TG 59:0	[M+Na]+	☑	☑
**986.9089**	986.911	2.1	ACer 63:3;O4	[M+H]+		☑
**997.9102**	997.9134	3.2	TG 60:0	[M+Na]+	☑	☑
**1009.7645**	1009.7621	2.4	TG 61:9	[M+K]+		☑
**1009.9125**	1009.9134	0.9	TG 61:1	[M+Na]+		☑
**1010.7756**	1010.7760	0.4	PS(50:0)	[M+Na]+		☑
**1011.7786**	1011.7777	0.9	TG 61:8	[M+K]+		☑
**1011.9263**	1011.9290	2.7	TG 61:0	[M+Na]+	☑	☑
**1012.7888**	1012.7916	2.8	PS(O-50:0(OH))	[M+Na]+		☑
**1025.7931**	1025.7934	0.3	TG 62:8	[M+K]+		☑
**1025.9415**	1025.9447	3.1	TG 62:0	[M+Na]+	☑	☑
**1033.8512**	1033.856	4.6	TG 62:4	[M+K]+		☑
**1034.7757**	1034.7760	0.3	PS(52:2)	[M+Na]+		☑
**1035.7786**	1035.7777	0.9	TG 63:10	[M+K]+		☑
**1036.7909**	1036.7916	0.7	PS(52:1)	[M+Na]+		☑
**1037.7951**	1037.7934	1.6	TG 63:9	[M+K]+		☑
**1037.9436**	1037.9447	1.1	TG 63:1	[M+Na]+		☑
**1038.8068**	1038.8073	0.5	PS(52:0)	[M+Na]+		☑
**1039.8101**	1039.809	1.1	TG 63:8	[M+K]+		☑
**1039.9567**	1039.9603	3.5	TG 63:0	[M+Na]+	☑	☑
**1041.6454**	1041.6485	3.0	PIM1 37:1	[M+H]+	☑	
**1045.8568**	1045.856	0.8	TG 63:5	[M+K]+	☑	
**1048.9057**	1048.9033	2.3	ACer 66:4;O3	[M+K]+	☑	

**1051.9589**	1051.9603	1.3	TG 64:1	[M+Na]+		☑
**1051.8093**	1051.809	0.3	TG 64:9	[M+K]+		☑
**1053.9723**	1053.976	3.5	TG 64:0	[M+Na]+	☑	☑
**1057.8515**	1057.856	4.3	TG 64:6	[M+K]+		☑
**1059.778**	1059.7777	0.3	TG 65:12	[M+K]+		☑
**1060.7907**	1060.7916	0.8	PS(54:3)	[M+Na]+		☑
**1061.7939**	1061.7934	0.5	TG 65:11	[M+K]+		☑
**1062.8067**	1062.8073	0.6	PS(54:2)	[M+Na]+		☑
**1062.9733**	1062.9763	2.8	ACer 68:3;O3	[M+Na]+	☑	
**1063.8101**	1063.809	1.0	TG 65:10	[M+K]+		☑
**1064.8227**	1064.8229	0.2	107899.4	[M+Na]+		☑
**1065.8233**	1065.8247	1.3	TG 65:9	[M+K]+	☑	☑
**1065.9753**	1065.976	0.7	TG 65:1	[M+Na]+		☑
**1066.8355**	1066.8386	2.9	PS(54:0)	[M+Na]+		☑
**1067.8367**	1067.8403	3.4	TG 65:8	[M+K]+	☑	
**1067.9882**	1067.9916	4.4	TG 65:0	[M+Na]+	☑	☑
**1077.8235**	1077.8247	1.1	TG 66:10	[M+K]+		☑
**1079.9891**	1079.9916	2.3	TG 66:1	[M+Na]+		☑
**1082.0025**	1082.0073	4.4	TG 66:0	[M+Na]+	☑	☑
**1088.8211**	1088.8229	1.7	PS(56:3)	[M+Na]+		☑
**1090.8374**	1090.8386	1.1	PS(56:2)	[M+Na]+		☑
**1091.0049**	1091.0076	2.5	ACer 70:3;O3	[M+Na]+	☑	
**1094.8642**	1094.8699	5.2	PS(56:0)	[M+Na]+		☑
**1096.0195**	1096.0229	3.1	TG 67:0	[M+Na]+	☑	
**1127.9509**	1127.9554	4.0	TG 66:1;O2	[M+K]+	☑	
**1151.4037**	1151.4036	0.1	PIP3 37:4	[M+K]+	☑	
**1162.3521**	1162.3499	1.9	CoA 24:5;O	[M+K]+		☑
